# Phlorizin, an Active Ingredient of *Eleutherococcus senticosus*, Increases Proliferative Potential of Keratinocytes with Inhibition of MiR135b and Increased Expression of Type IV Collagen

**DOI:** 10.1155/2016/3859721

**Published:** 2016-03-06

**Authors:** Hye-Ryung Choi, Kyung-Mi Nam, Hyun-Sun Lee, Seung-Hye Yang, Young-Soo Kim, Jongsung Lee, Akira Date, Kazumi Toyama, Kyoung-Chan Park

**Affiliations:** ^1^Department of Dermatology, Seoul National University Bundang Hospital, Seongnam, Gyeonggi 463-707, Republic of Korea; ^2^Biospectrum Life Science Institute, Seongnam, Gyeonggi 462-807, Republic of Korea; ^3^Department of Genetic Engineering, Sungkyunkwan University, 2066 Seobu-ro, Suwon City, Gyunggi Do 164-19, Republic of Korea; ^4^Department of Medical Pharmacy, Hyogo University of Health Sciences, Kobe 650-8530, Japan; ^5^P&G Group, Kobe 658-0032, Japan

## Abstract

*E. senticosus* extract (ESE), known as antioxidant, has diverse pharmacologic effects. It is also used as an antiaging agent for the skin and phlorizin (PZ) is identified as a main ingredient. In this study, the effects of PZ on epidermal stem cells were investigated. Cultured normal human keratinocytes and skin equivalents are used to test whether PZ affects proliferative potential of keratinocytes and how it regulates these effects. Skin equivalents (SEs) were treated with ESE and the results showed that the epidermis became slightly thickened on addition of 0.002% ESE. The staining intensity of p63 as well as proliferating cell nuclear antigen (PCNA) is increased, and integrin *α*6 was upregulated. Analysis of ESE confirmed that PZ is the main ingredient. When SEs were treated with PZ, similar findings were observed. In particular, the expression of integrin *α*6, integrin *β*1, and type IV collagen was increased. Levels of mRNA for type IV collagen were increased and levels of miR135b were downregulated. All these findings suggested that PZ can affect the proliferative potential of epidermal cells in part by microenvironment changes via miR135b downregulation and following increased expression of type IV collagen.

## 1. Introduction

*Eleutherococcus senticosus (Acanthopanax senticosus)*, known as Siberian ginseng or ciwujia, is a kind of small woody shrub which belongs to the family Araliaceae. It has diverse pharmacological effects, including antifatigue, learning improvement, anti-inflammatory, immune-enhancing, and antidepressive effects [1]. It has protective effect against oxidative damage, and it has been used as a skin antiaging agent [2].

Skin is maintained by epidermal stem cells that self-renew, proliferate, and differentiate [3, 4]. Therefore, skin aging is induced by impaired stem cell mobilization or by a reduction in the number of stem cells that can respond to proliferative signals [5]. Self-renewal of stem cells is controlled intrinsically by gene expression and it is modulated through interaction with extrinsic cues from the environment [6]. Thus, modulation of the stem-cell niche is important for the self-renewal and multipotency of stem cells [7]. It is reported that low oxygen tensions (hypoxia) are necessary to maintain undifferentiated stem cell phenotypes and also influence proliferation and stem cell fate [8]. We also reported that redox status is critical for stem cell activities [9]. It is well known that ESE has antioxidant activity [2] and PZ is found to be the main ingredient and it has antioxidant activity [10]. It has also been reported that PZ protects skin against UVB-induced skin damage by decreasing ROS overproduction [11]. These findings suggested that PZ may have beneficial effects on skin stem cells. In the current study, we investigated the effects of an extract of* E. senticosus* (ESE) and its active ingredient, phlorizin (PZ) {3,5-dihydroxy-2-[3-(4-hydroxyphenyl)propanoyl]phenyl *β*-d-glucopyranoside}, on human epidermal cells. Results showed that PZ recovered proliferative potential of epidermal cells by affecting basement membrane which constitute niche of epidermal basal cells. Recently, we reported that type IV collagen is a target of miR135b and that miR135b suppression may improve the microenvironment and also increase the proliferative potential of epidermal basal cells [12]. In this study, the effect of PZ was investigated in terms of miR135b and type IV collagen expression.

## 2. Materials and Methods

### 2.1. Reagents

Unless otherwise specified, reagents including phlorizin (Phlorizin, P3449) were obtained from Sigma-Aldrich. The antibodies that we used in this study were as follows: p63 antibody (#sc-8431, Santa Cruz Biotechnology, Santa Cruz, CA), proliferating cell nuclear antigen antibody (PCNA, #M0879, DAKO, Glostrup, Denmark), integrin *α*6 antibody (#sc-6597, Santa Cruz Biotechnology), integrin *β*1 antibody (#sc-9970, Santa Cruz Biotechnology), and type IV collagen antibody (239M-16, Cell Marque, Rocklin, CA).

### 2.2. Preparation of* E. senticosus* Extracts and Identification of Phlorizin

Air-dried powdered roots (100 g) of* E. senticosus* were extracted with 80% aqueous methanol (1000 mL) at room temperature. The 80% aqueous methanol extract was concentrated under reduced pressure and then lyophilized to yield 21 g of dry extract, which was stored at −20°C. Concentrated extract was suspended in water. The extract was then fractionated successively with equal volumes of hexane, chloroform, ethyl acetate, and butan-1-ol. The ethyl acetate fraction was further purified by reversed-phase chromatography (LiChroprep RP-C18, Merck). The EtOAc fraction was dissolved in 10% MeOH (100 mL) and subjected to open-column chromatography. Elution was carried out with aqueous MeOH increasing the MeOH content in 10% increment from 10% to 100% (100 mL each). The 30% aqueous MeOH fraction was concentrated in vacuo. The active compound was then separated by preparative reversed-phase HPLC [Luna C18 (2) column, 21.2 × 250 mm, 5 *μ*m, Phenomenex]. The mobile phase was a 45-min linear methanol-water gradient (5 : 95 to 95 : 5) flowing at a rate of 24 mL/min. To identify the active ingredient of ESE, we examined the effects of the various HPLC fractions by means of MTT assay. The active ingredient was found to be present in HPLC fractions 32–36. HPLC fractions 32–36 were combined and concentrated in vacuo (13 mg), and the chemical structure of the isolated product was determined by examining its 1H- and 13C-NMR spectra (Bruker Avance-500, 500 MHz).

### 2.3. DPPH Radical Scavenging Assay

A sample of each stock solution (2 *μ*L, 10 mg/mL) was added to 80 *μ*L of 0.25 mM 1,1-diphenyl-2-picrylhydrazyl (DPPH) and 118 *μ*L of 70% ethanol to give a final DPPH concentration of 0.1 mM. Next, the mixture was vigorously shaken and then left to stand for 10 min in the dark. The absorbance at 517 nm was then measured using ELISA reader (TECAN, Salzburg, Austria) [13].

### 2.4. Cell Culture and Toxicity Assay

Normal human fibroblasts and keratinocytes were isolated from human foreskins obtained during circumcision following our previous protocol [9]. All samples were obtained with informed consent. For culture of keratinocytes or fibroblast, keratinocyte growth medium (KGM, Clonetics, San Diego, CA) or Dulbecco's modified Eagle's medium (DMEM) (LM001-05, WelGENE, Daegu, South Korea) supplemented with 10% fetal bovine serum (FBS, Thermo Scientific HyClone, Logan, UT) was used. The medium was changed every two or three days. For keratinocytes, 4 × 10^4^ cells suspended in KGM were seeded into each well of a 24-well plate. After 24 h, the medium was replaced with keratinocyte basal medium (KBM, Clonetics) supplemented with 0.1% bovine serum albumin (BSA). For fibroblasts, cells were also incubated for 24 h in FBS-free DMEM before treatment. ESE or PZ was added and the cells were incubated for further 24 h at 37°C. A 5 mg/mL solution of 2-(4,5-dimethyl-1,3-thiazol-2-yl)-3,5-diphenyl-2*H*-tetrazol-3-ium bromide (MTT) in DPBS (100 *μ*L) was mixed and the plates were incubated for 4 h. After removing the supernatant, the formazan crystals were dissolved in 1 mL of dimethyl sulfoxide. The optical density was then determined at 540 nm by using ELISA reader (Tecan Austria GmbH, Grödig, Austria).

### 2.5. Construction of Skin Equivalents

Skin equivalents (SEs) were prepared by our method [14]. Type I collagen was extracted from the tendons of rat tails. Collagen substitutes were prepared as follows: eight volumes of type I collagen with one volume of 10x concentrated DMEM and one volume of neutralization buffer (0.05 M NaOH, 0.26 mM NaHCO_3_, and 200 mM 2-[1-(2-hydroxyethyl)piperazin-1-ium-4-yl]ethanesulfonate) (HEPES) and then adding 5 × 10^5^ fibroblasts. After gelling (3.0 *μ*m Millicell, Millipore, Bedford, MA), keratinocytes (1 × 10^6^ cells) were then seeded onto the surface of collagen substitute. After 1 day in a submerged state, they were maintained at the air-liquid interface for additional 12 days. The growth medium consists of DMEM and Ham's nutrient mixture F12 in a ratio of 3 : 1, supplemented with 5% FBS, 0.4 *μ*g/mL hydrocortisone, 1 *μ*M isoproterenol, 25 *μ*g/mL ascorbic acid, and 5 *μ*g/mL insulin. Every week, the medium was changed for 3 times. All experiments were repeated at least twice under the same conditions. Samples were treated with the medium for 7 days before harvesting the SEs.

### 2.6. Histology and Immunohistochemistry

Finally, the SEs were fixed with Carnoy's fixative and embedded in paraffin. To stain for type IV collagen, antigen retrieval was performed by using proteinase K (Roche Applied Science, Penzberg, Germany). For other antibodies, thermal antigen retrieval was performed by using Trilogy solution (Cell Marque) and a pressure cooker. Immunohistochemical staining was performed by using the avidin-biotin-peroxidase complex technique (DAKO). All experiments were repeated twice. For image analysis, same condition was applied for staining and taking pictures. After taking pictures, expression of type IV collagen and integrins was measured by stained areas in three representative areas. In case of PCNA (proliferating cell nuclear antigen) and p63, expression was measured by OD (optical density: intensity/stained area) in three representative areas. Images were analyzed quantitatively using MetaMorph Offline version 7.7.0.0 image analysis software (Molecular Devices, Downingtown, PA). To see the effects of phlorizin on fibroblasts, SEs were stained with Hoechst 33342 (1 *μ*g/mL, 40047, Biotium Inc., Hayward, CA, USA) for 10 min at RT. Then number of dermal cells in 3 representative areas was compared.

### 2.7. RT-PCR Analysis

One hundred thousand of keratinocytes were seeded in well of 6-well plate. The cells were cultured in phlorizin (0, 50, 100, and 200 *μ*M) containing media for 72 hrs. Total RNA was isolated using AllPrep DNA/RNA/Protein Mini Kit (80004, Qiagen, Valencia, CA). Quality and amount of the RNA were assessed using Experion RNA StdSens analysis kit (700-7104, Bio-Rad, Hercules, CA) by Experion automated electrophoresis system (700-7001, Bio-Rad). For the qRT-PCR of human COL4A3, ITGA6, and ITGB1, one microgram of total RNA was reverse transcribed into cDNA (ImProm-II Reverse Transcription System, A3800, Promega, Madison, WI) with Oligo(dT) 15 Primer following manufacturer's instructions. Real-time PCR was performed with TaqMan Gene Expression Master Mix (4369016, Applied Biosystems, Foster City, CA) and target gene primers: COL4A3 (4331182, Hs01022542_m1, Applied Biosystems), ITGA6 (4331182, Hs01041011_m1, Applied Biosystems), and ITGB1 (4331182, Hs00559595_m1, Applied Biosystems). The housekeeping gene, GAPDH (43352934E, Applied Biosystems), was used as an endogenous control. For qRT-PCR of has-miR135b, 10 ng of total RNA was reverse transcribed into cDNA using TaqMan MicroRNA Reverse Transcription Kit (4366596, Applied Biosystems). Real-time PCR was performed with TaqMan Universal PCR Master Mix II (4440038, Applied Biosystems, Foster City, CA) and TaqMan MicroRNA Assays (4427975, 002261, Applied Biosystems) specific for has-miR135b on ViiA 7 Real-Time PCR System (Applied Biosystems). RNU6B (4427975, 001093, Applied Biosystems) was used as an endogenous control.

### 2.8. Statistical Analysis

Data were compared by using a Mann-Whitney test (PASW Statistics 18, PASW, Chicago, IL).

## 3. Results

### 3.1. The Effects of ESE on LSE

**Figure 1:**
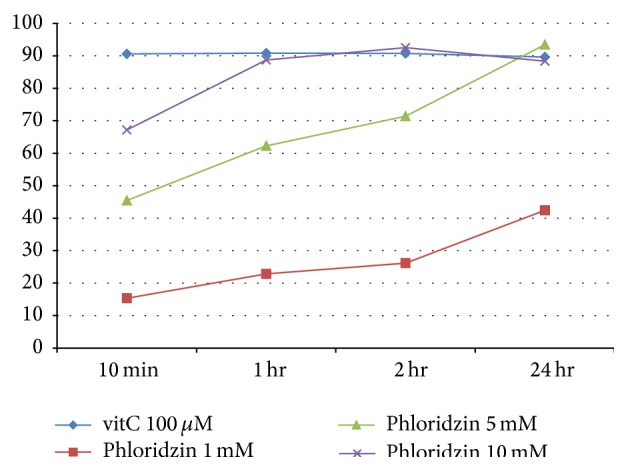
DPPH assay of vitamin C and phlorizin.

**Figure 2:**
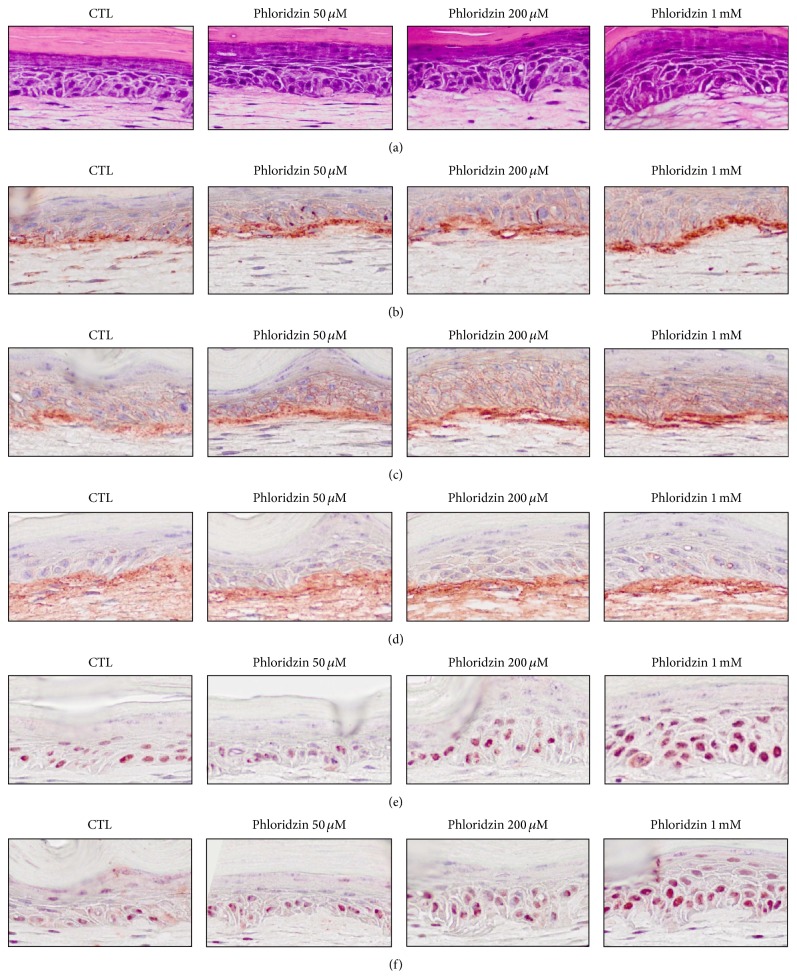
Histologic findings for PZ-treated SEs. SEs were constructed and then incubated in the presence of PZ (0, 50, and 200 *μ*M or I mM). Sections of SEs were stained with hematoxylin and eosin and analyzed by immunohistochemical staining ((a): H&E staining, (b): integrin *α*6, (c): integrin *β*1, (d): type IV collagen, (e): p63, and (f): PCNA).

**Figure 3:**
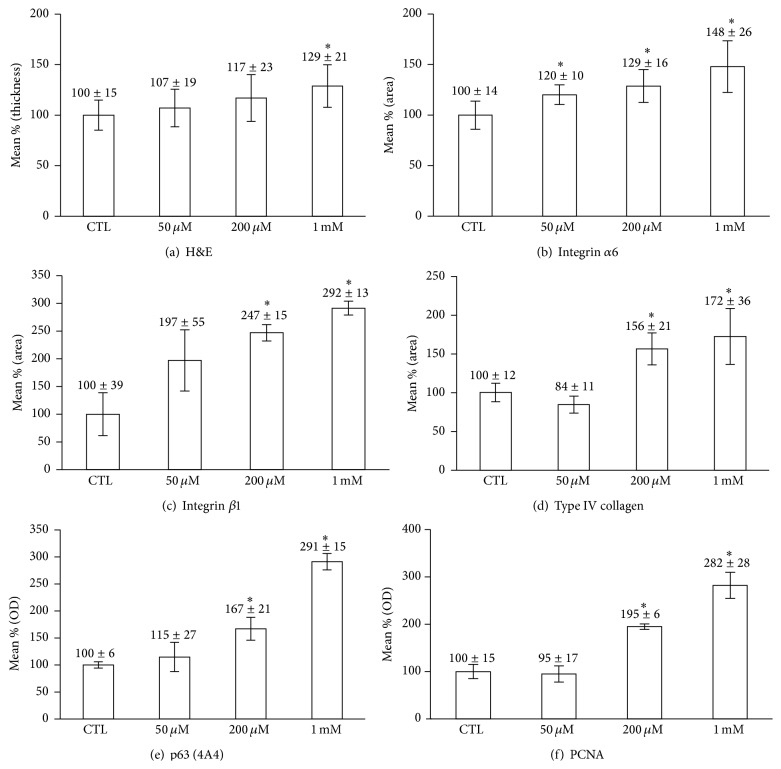
Comparison of epidermal thickness, staining intensity, and the numbers of p63- and PCNA-positive cells. Immunohistochemical staining was analyzed quantitatively by using Image J software (National Institute of Health, Bethesda, Maryland, USA). The positivity of p63 and PCNA was measured as described in Section 2. ^*∗*^ *P* < 0.05 compared to contro.

Cells were treated with ESE. The results showed that ESE was slightly toxic at a concentration of 0.005% (S-Figure  1a, 1b, in Supplementary Material available online at http://dx.doi.org/10.1155/2016/3859721). To confirm these effects, different fraction was tested and similar results were obtained (S-Figure  1c, 1d). We therefore chose concentrations of 0.001% and 0.002% for LSE construction. In all the SE models, well stratified epidermis was observed and the epidermis became slightly thickened on treatment with a higher concentration (0.002%) of ESE (S-Figures  2 and 3). Integrin *α*6, extracellular adhesion receptors, is located along the dermal epidermal junction and it means the presence of hemidesmosomes [15]. Intense and linear staining of *α*6 integrin was observed along the basement membrane in ESE models (S-Figures  2 and 3). p63 is known as a stem cell marker that belongs to the p53 family of genes and it is expressed in basal and suprabasal layers of the epidermis [16]. ESE-treated SEs showed increased intensity of p63 compared with control SEs (S-Figures  2 and 3). Expression of PCNA [17] is observed in the basal layer and there was increased intensity in PCNA staining in the ESE-treated models (S-Figures  2 and 3). All experiments were repeated twice.

### 3.2. Identification of Phlorizin

**Figure 4:**
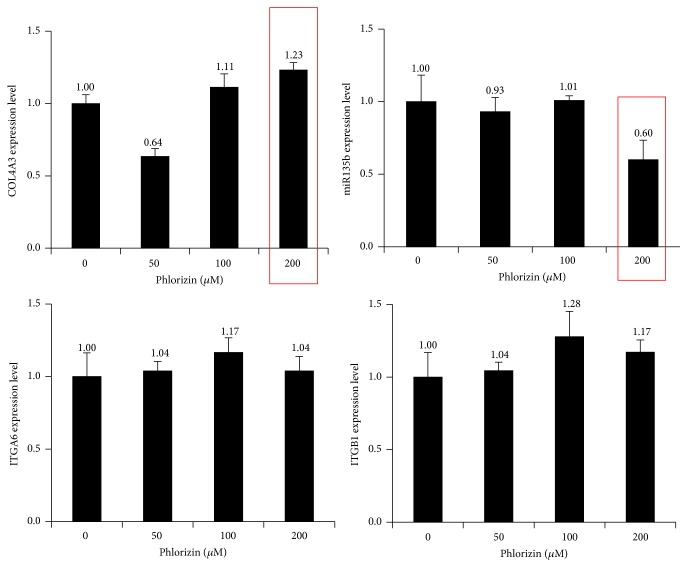
RT-PCR analysis of miR135b and mRNA of integrins and type IV collagen. Keratinocytes were treated with increasing doses of PZ and total RNA was extracted and reverse transcribed into cDNA. Real-time PCR was performed as described in Section 2.

To identify the active ingredient of ESE, we examined the effect of various fractions from an aqueous methanolic extract of ESE. For proliferation assay of keratinocytes, full medium was used by means of MTT assay (data not shown). The chemical structure of the purified compound from* E. senticosus* was determined. The 1H NMR spectrum showed the presence of two mutually coupled doublets (*δ* = 5.95 and 6.15, *J* = 1.9 Hz) that are characteristic of an unsymmetrically substituted phloroglucinol ring, a quartet (*δ* = 6.65 and 7.01, *J* = 8.4 Hz) corresponding to protons of the* para*-disubstituted benzene ring and a multiplet (*δ* = 3.41) and a triplet (*δ* = 2.85, *J* = 7.6 Hz) attributable to methylene protons adjacent to a ketone structure. In addition, the 1H NMR spectrum also exhibited signals arising from a *β*-glucopyranosyl unit (*δ* = 5.01, *J* = 7.0 Hz) and a sugar moiety (*δ* = 3.08–3.71). The 13C NMR spectrum showed signals for 21 carbon atoms. Analysis of COSY, HMQC, and HMBC data showed that these NMR features were consistent with the structure of phlorizin {3,5-dihydroxy-2-[3-(4-hydroxyphenyl)propanoyl]phenyl *β*-d-glucopyranoside} (S-Figure  4).

1H NMR (500 MHz, CD3OD): *δ* = 2.85 (t, *J* = 7.6 Hz, 2H, H-9), 3.08 (m, 1H, H-4′′), 3.10 (m, 1H, H-5′′), 3.15 (m, 1H, H-2′′), 3.17 (m, 1H, H-3′′), 3.41 (m, 2H, H-8), 3.50 (dd, *J* = 5.1 and 11.9 Hz, 1H, H-6′′), 3.71 (d, *J* = 11.9 Hz, 1H, H-6′′), 5.01 (d, *J* = 7.0 Hz, 1H, H-1′′), 5.95 (d, *J* = 1.9 Hz, 1H, H-5), 6.15 (d, *J* = 1.9 Hz, 1H, H-3), 6.65 (d, *J* = 8.4 Hz, 2H, H-2′ and H-6′), 7.01 (d, *J* = 8.4 Hz, 2H, H-3′ and H-5′). 13C NMR (125 MHz, CD3OD): *δ* = 30.8 (t, C-9), 46.9 (t, C-8), 62.2 (t, C-6′′), 71.3 (d, C-4′′), 74.6 (d, C-2′′), 78.1 (d, C-5′′), 78.3 (d, C-3′′), 95.2 (d, C-5), 98.3 (d, C-3), 102.1 (d, C-1′′), 106.8 (s, C-1), 115.8 (d, C-2′ and C-6′), 130.2 (d, C-3′ and C-5′), 133.6 (s, C-1′), 56.3 (s, C-4′), 162.2 (s, C-4), 165.6 (s, C-6), 167.5 (s, C-2), 206.5 (s, C-7).

## 4. Antioxidant Activity of PZ

Vitamin C was chosen as a control. Scavenging activity was tested by DPPH radical scavenging assay. Compared to vitamin C, PZ showed relatively weak antioxidant activity at low concentrations but good antioxidant activities at high concentrations (10 mM) (Figure 1).

### 4.1. Cytotoxicity of PZ

The cytotoxicity of PZ was analyzed by MTT assay, in which normal human keratinocytes and fibroblasts were treated with PZ. PZ was not toxic at any of the concentrations tested up to 200 *μ*M (S-Figure  5). To test the proliferative effects on keratinocytes, normal human keratinocytes were treated for 72 hr and results showed that PZ is not stimulatory at any concentrations (S-Figure  6). The effects of PZ were then tested at concentrations of 50 *μ*M to 1 mM in LSE.

### 4.2. Histology of PZ-Treated SEs

The presence of a stratified epidermis was observed in all the models. PZ increased the thickness of the epidermis (Figures 2 and 3). To see the effects on fibroblast, Hoechst 33342 stained cells were counted in 3 different parts of dermal portion in SEs and there was no significant difference according to the concentration of PZ (S-Figure  7).

### 4.3. Immunohistochemical Staining of PZ-Treated SEs

Integrin *α*6 is a marker of extracellular adhesion receptors [15] and integrin *β*1, expressed throughout the basal cell membrane, is important in cell-matrix and cell-cell interactions [18]. Integrins *α*6 and *β*1 were observed along the basement membrane, and the staining of both integrins *α*6 and *β*1 was significantly increased at dose-dependent manner (Figures 2 and 3). In addition, type IV collagen, a major component of basement membrane [19], was stained. Linear staining of type IV collagen increased in a dose-dependent manner in the PZ-treated models (Figures 2 and 3). p63 and PCNA expression were also increased in a dose-dependent manner (Figures 2 and 3). Experiments were repeated at least twice and the results are representative data from repeated experiments.

### 4.4. RT-PCR Analysis for miR135b and mRNA of Integrins and Type IV Collagen

Results showed that levels of miR135b were decreased and expression of type IV collagen mRNA was increased. But mRNA of integrin *α*6 and integrin *β*1 was not significantly changed (Figure 4).

## 5. Discussion

*E. senticosus* has similar herbal properties to* Panax* ginseng. We found that PZ is the main ingredient of ESE and the effect of PZ on human epidermal cells was tested.

Redox balance is important determinant of stem cell fate [9] and ESE as well as PZ is known to have antioxidant activity [2]. Thus, the effects of PZ were tested in terms of stem cell fate in the skin.

Epidermis is the outermost layer of the skin and needs to self-renew and regenerate continuously. This ability is dependent on keratinocyte which can proliferate and respond to differentiation cues [20]. ESE definitely increased the intensity of p63 staining compared with control SEs and it means that ESE increases the proliferative potential of epidermal basal cells. The PCNA staining also showed increased intensity in ESE-treated SEs (S-Figures  2 and 3). Extraction and chromatography showed that PZ is an active ingredient of ESE. Then, the effects of PZ were tested by similar pattern of experiments. PZ increased the thickness of the epidermis (Figures 2 and 3). Furthermore, PZ is not stimulatory to keratinocytes proliferation at any concentrations tested (S-Figure  6) in monolayer culture. This suggests that PZ may increase the proliferative potential of epidermal cells by indirect mechanism. Epidermal homeostasis depends on a balance between renewal and differentiation of stem cells and it is regulated by extrinsic signals from the extracellular matrix (ECM) [21]. The status of the basement membrane was stained. Staining of integrin *α*6, integrin *β*1, and type IV collagen increased along the basement membrane, and the staining intensity increased dose dependently (Figures 2 and 3). We then stained for p63 and PCNA. The results clearly showed that the intensity of p63 and PCNA staining are also increased in a dose-dependent manner (Figures 2 and 3). These findings suggest that PZ can affect the stem cell characteristics and proliferative potential of epidermal cells. As far as we know, this is the first report of the fact that PZ, a key component of ESE, might affect the stem cell characteristics of keratinocytes by regulating extracellular matrix proteins or transmembrane protein such as type IV collagen and integrins. Because PZ also may affect epidermal cells through fibroblasts, the number of dermal cells was compared in SEs. But there were no significant differences according to the concentration of PZ (S-Figure  6). These finding suggested that PZ may not affect keratinocytes through fibroblasts.

It is described that the behavior of all stem cells is controlled by the interplay between intrinsic transcriptional programs and extrinsic signals [21]. Recently we also reported that oligosaccharides of hyaluronic acid and various antioxidants can affect the stem cell character and proliferative potential of epidermal basal cells by providing more favorable microenvironment [9, 22]. PZ inhibits polycystic ovary disease progression by targeting sodium-glucose cotransporter [23]. Furthermore, the calcium-dependent desmosome formation was affected by the sodium-regulated keratinization in frog skin cultures [24]. In addition, human keratinocytes stem cells survive for months in sodium chloride [25]. Thus, PZ may regulate keratinocytes by targeting sodium-glucose transport. Interestingly, it is also reported that there is a relationship between sodium chloride concentration and free radical scavenging activity [26]. Cell fate is regulated by the expression of specific genes [27] and microRNAs (miRNAs) are regulators of gene expression. Recently, we found that type IV collagen is a target of miR135b and that miR135b suppression improved the microenvironment and increase the proliferative potential of basal cells [12]. It disclosed a role of miR135b in epidermal keratinocytes and may provide a way to control stem cell fate in the skin. All these findings showed that topical application of ESE or PZ may have antiaging effects on the skin through miR135b-type IV collagen pathway. Thus, miR and extracellular matrix mediated pathway can be an important antiaging strategy.

In this study, PZ increased the staining intensity of *α*6 integrin, *β*1 integrin, and type IV collagen which is associated with increased number of p63 and PCNA positive cells. RT-PCR also showed that type IV collagen mRNA was increased but the expression levels of both integrins *α*6 and *β*1 mRNA was not changed. The reason of these differences may be derived from different models but needs further extensive study. Furthermore, level of miRNA135b was decreased by PZ treatment. Type IV collagen is a main scaffold of basement membrane. Thus, all these findings suggested that PZ can affect the niche condition by increased expression of type IV collagen.

## 6. Conclusions

It can be concluded that PZ, which has antioxidant activity, can affect stem cell fate in the skin via inhibition of miR135b and following synthesis of type IV collagen of basement membrane.

## Supplementary Material


*Eleutherococcus senticosus* (*Acanthopanax senticosus*) has protective effect against oxidative damage and it has been used as a skin anti-aging agent. *E. senticosus* extracts (ESE) was prepared and toxicity was tested against cultured normal human keratinocytes and fibroblasts (S-Figure 1). The results showed that ESE was slightly toxic to both keratinocytes and fibroblasts at a concentration of 0.005%. Cultured skin equivalents (SEs) were treated with ESE and results showed the beneficial effect by ESE (S-Figure 2, S-Figure 3). In all the SE models, well stratified epidermis was observed and the epidermis became slightly thickened on addition of higher concentration (0.002%) of ESE. Intense and linear staining of α6 integrin was also observed at higher concentration. Interestingly, immunohistochemical staining showed increased intensity of p63 and PCNA in the ESE-treated models. ESE was purified and phlorizin (PZ) was found to be a main ingredient (S-Figure 4). Compared to ESE, PZ was not toxic to cultured normal human keratinocytes and fibroblasts (S-Figure 5). To check the possibility of direct effects on keratinocytes, the effects of PZ on cultured human keratinocytes were tested. Results showed that PZ is not stimulatory to cultured human keratinocytes (S-Figure 6). The effects of PZ on fibroblasts were also tested and results showed that PZ also didn't show effects on fibroblasts in dermal part of SE (S-Figure 7).
