# Supplementary material for: Phlorizin, an Active Ingredient of Eleutherococcus senticosus, Increases Proliferative Potential of Keratinocytes with Inhibition of MiR135b and Increased Expression of Type IV Collagen
Source: Oxid Med Cell Longev. 2016 Mar 6;2016:3859721. doi: 10.1155/2016/3859721 (PMC4799823; doi:10.1155/2016/3859721)
Supplement: Supplementary file 1 — Eleutherococcus senticosus (Acanthopanax senticosus) has protective effect against oxidative damage and it has been used as a skin anti-aging agent. E. senticosus extracts (ESE) was prepared and toxicity was tested against cultured normal human keratinocytes and fibroblasts (S-Figure 1). The results showed that ESE was slightly toxic to both keratinocytes and fibroblasts at a concentration of 0.005%. Cultured skin equivalents (SEs) were treated with ESE and results showed the beneficial effect by ESE (S-Figure 2, S-Figure 3). In all the SE models, well stratified epidermis was observed and the epidermis became slightly thickened on addition of higher concentration (0.002%) of ESE. Intense and linear staining of α6 integrin was also observed at higher concentration. Interestingly, immunohistochemical staining showed increased intensity of p63 and PCNA in the ESE-treated models. ESE was purified and phlorizin (PZ) was found to be a main ingredient (S-Figure 4). Compared to ESE, PZ was not toxic to cultured normal human keratinocytes and fibroblasts (S-Figure 5). To check the possibility of direct effects on keratinocytes, the effects of PZ on cultured human keratinocytes were tested. Results showed that PZ is not stimulatory to cultured human keratinocytes (S-Figure 6). The effects of PZ on fibroblasts were also tested and results showed that PZ also didn't show effects on fibroblasts in dermal part of SE (S-Figure 7). [file 3859721.f1.doc]

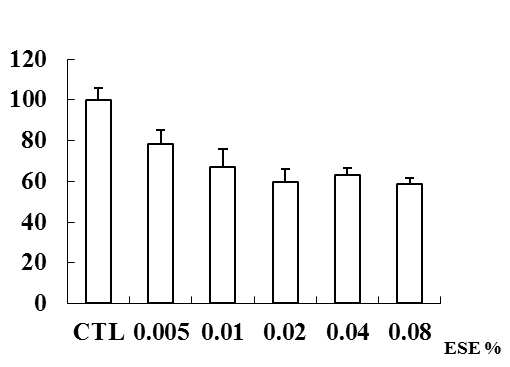

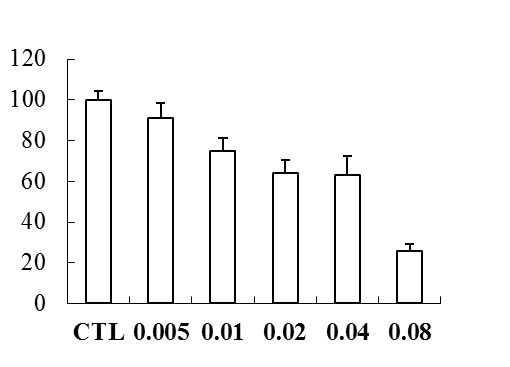

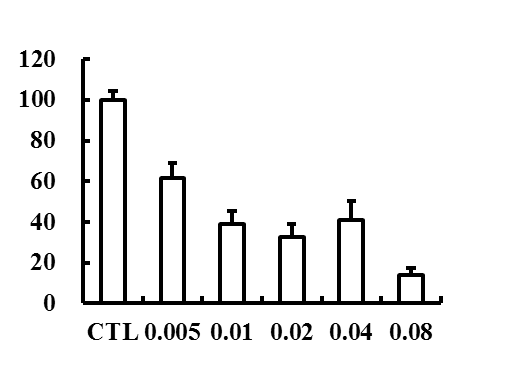


**a) KC cytotoxicity**

**c) KC cytotoxicity**

**b) FB cytotoxicity**

**d) FB cytotoxicity**


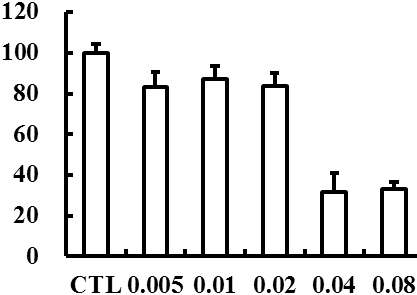


S-Fig 1. MTT assay of keratinocytes (a, c) and fibroblasts (b, d) after incubation for 24 h at 37 °C under 5% CO_2_ in the presence of various concentrations of *E. senticosus* extracts (ESE). ESE was prepared and toxicity was tested against cultured normal human keratinocytes and fibroblasts. ESE was slightly toxic to both keratinocytes and fibroblasts at a concentration of 0.005%. The results show the MTT value as a percentage of that of the control. The values are the means ± S.D. for triplicate wells.

**
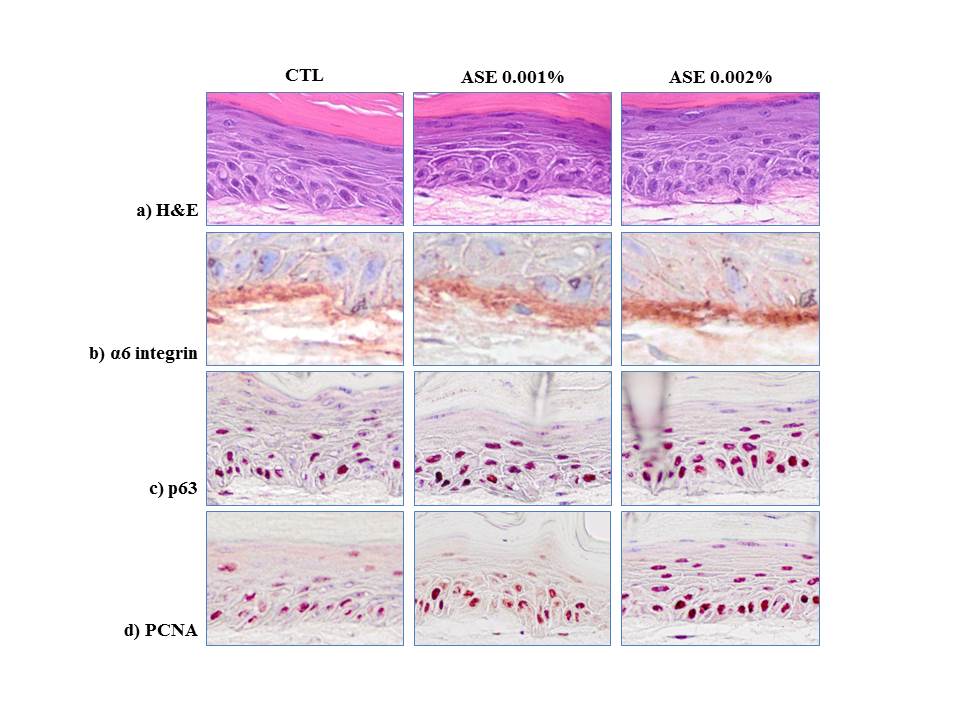
**

S-Fig 2. Histologic findings of ESE-treated SEs. SEs were constructed and then incubated in the presence of 0.001% or 0.002% of ESE. Sections of SEs were stained with hematoxylin and eosin, and analyzed by immunohistochemical staining (a: H&E staining, b: integrin α6, c: p63, d: PCNA). In all the SEs, well stratified epidermis was observed and the epidermis became slightly thickened on addition of higher concentration of ESE (0.002%). Intense and linear staining of α6 integrin was also observed at higher concentration. Increased intensity of p63 and PCNA was also observed in the ESE-treated models. The experiment was repeated twice and representative data are shown.


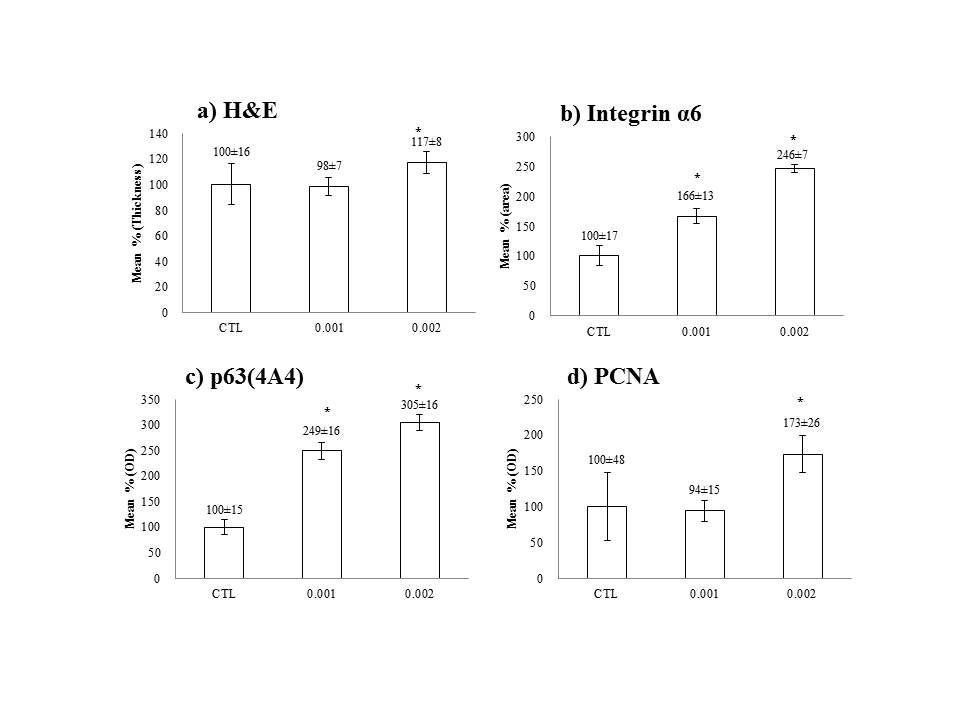


S-Fig 3. Comparison of epidermal thickness and staining intensity in ESE-treated SEs. Immunohistochemical staining was analyzed quantitatively. The staining intensity was evaluated as described in the materials and methods.


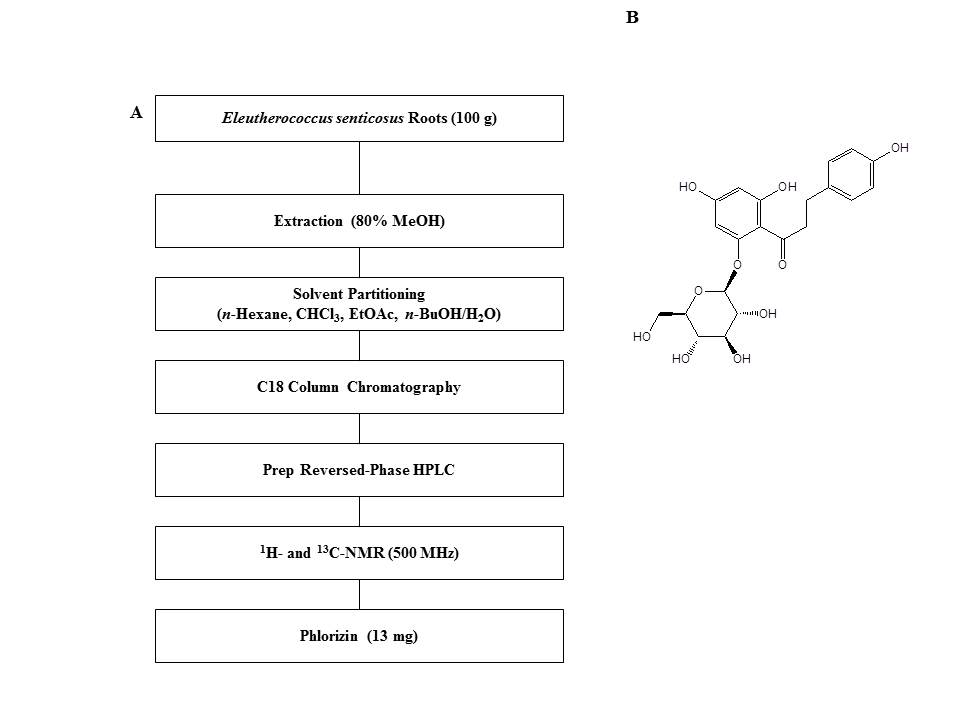


S-Fig 4. Identification and the chemical structure of phlorizin. Phlorizin (PZ) was found to be a main ingredient.


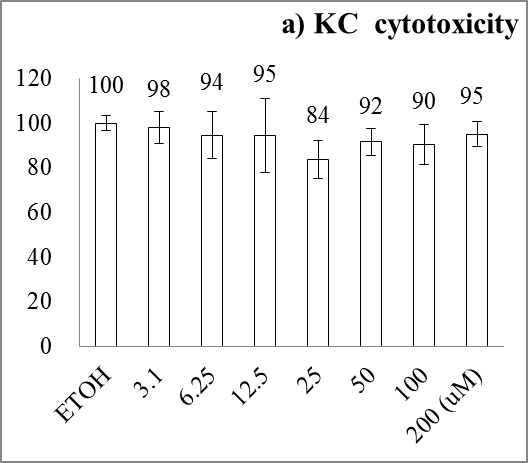

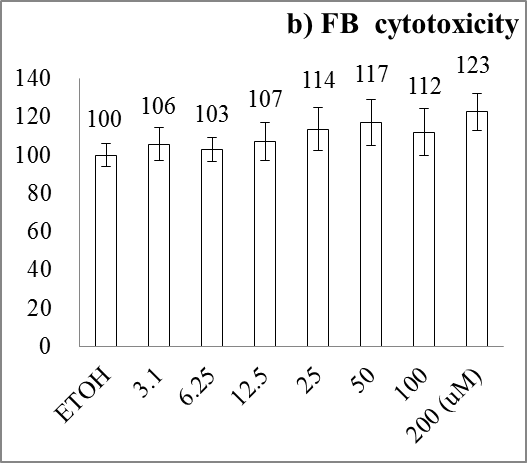


S-Fig 5. MTT assay of keratinocytes (a) and fibroblasts (b) after incubation for 24 h at 37 °C under 5% CO_2_ in the presence of increasing concentrations of PZ. PZ was not toxic to cultured normal human keratinocytes and fibroblasts. The results show the MTT value as a percentage of that of the control. Values are the means ± S.D. for triplicate wells

1. b)


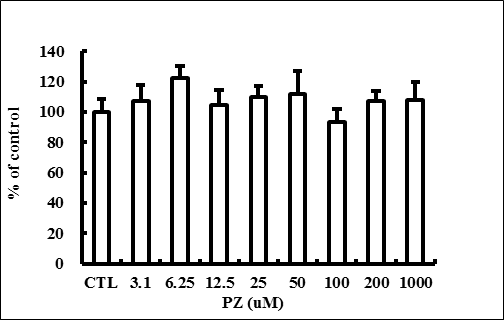

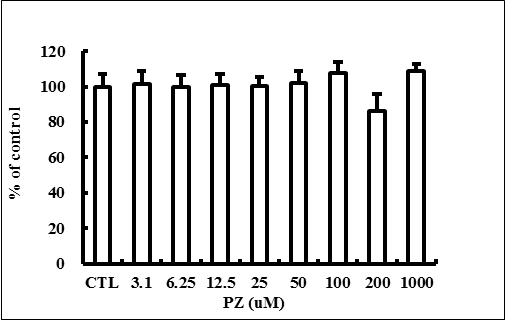


S-Fig 6. MTT assay of keratinocytes (a, b) after incubation for 72 h at 37 °C under 5% CO_2_ in the presence of increasing concentrations of PZ. PZ is not stimulatory to cultured human keratinocytes. The results show the MTT value as a percentage of that of the control. Values are the means ± S.D. for triplicate wells

**
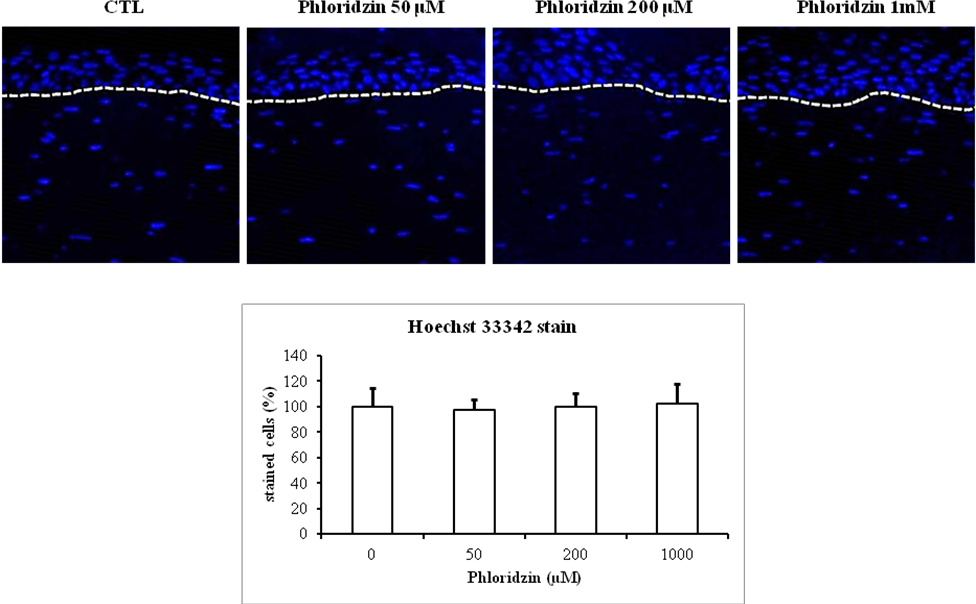
**

S-Fig. 7. The effects of PZ on fibroblast. a) Hoechst 33342 stainining in SEs. b) Stained cells were counted in dermal part of SEs. Results showed that PZ didn’t show any effects on fibroblasts in dermal part of SE. The values are the means ± S.D compared to control.
